# Supplementary material for: Identification of Freezing-Responsive microRNAs and Their Targets in Chinese Jujube by Small RNA and Degradome Sequencing
Source: Int J Mol Sci. 2026 Sep 4;27(17):7912. doi: 10.3390/ijms27177912 (PMC13566890; doi:10.3390/ijms27177912)
Supplement: Supplementary file 1 [file ijms-27-07912-s001.zip › Supplementary Material S2/novel_miR_46.pdf]

Provisional ID	:	novel_miR_46
Score total	:	284.2
Score for star read(s)	:	3.9
Score for read counts	:	275.4
Score for mfe	:	3.2
Score for randfold	:	1.6
Score for cons. seed	:	
Total read count	:	552
Mature read count	:	275
Loop read count	:	0
Star read count	:	277

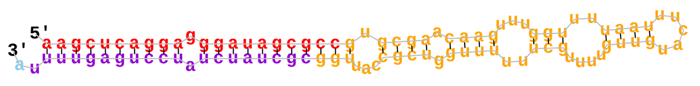

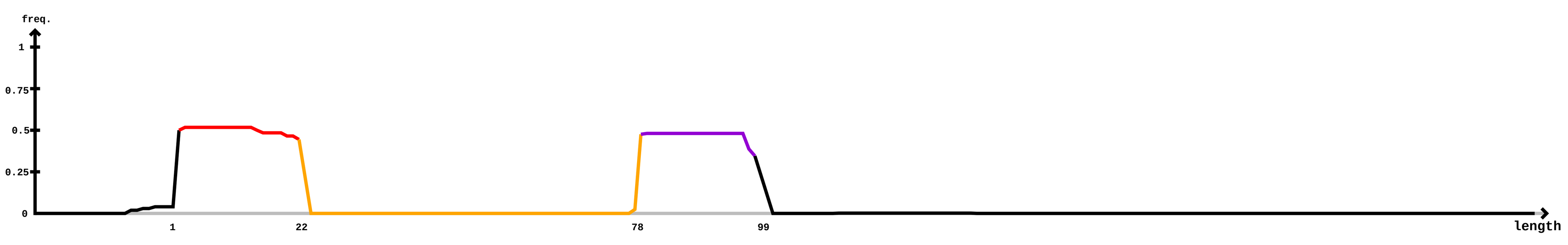

Mature Star

```
5'- aaacaguaagugagaaucuguaaagcucaggagggauagcgccgugcgaacaaguuugguuuuaauuucauguuguuuugcuuuuuuggucgccauuggcgcuaucuauccugaguuuuauagguucuucuuacuacauguauaucucugguacucaugcaguacaguuuucaguucacucagcuggagguauuguauuuggcuccuugguaugcaacaugauacuacauauaucuguuaaguaauuuac -3'  obs
    aaacaguaagugagaaucuguaaagcucaggagggauagcgccgugcgaacaaguuugguuuuaauuucauguuguuuugcuuuuuuggucgccauuggcgcuaucuauccugaguuuuauagguucuucuuacuacauguauaucucugguacucaugcaguacaguuuucaguucacucagcuggagguauuguauuuggcuccuugguaugcaacaugauacuacauauaucuguuaaguaauuuac       exp
    ....(((((.((((((((((((((((((((.((((((((((.((((.((((...(((..((((.....))))....)))..)))).))))...))))))))))).)))))))))))))))))))))))))...((((((((...((..((.(((((((((...((((((((.....))))))))))))))))..))..))..))))))))(((.((((.......))))))).........  reads  mm  sample
    ..............aaucuguaaagcucaggaggg...            8   0   JCK-2
    ..............aaucuguaaagcucaggaggga...           3   0   LT-1
    ................ucuguaaagcucaggaggg...            2   0   JCK-3
    ................ucuguaaagcucaggagggauagc...       2   0   JCK-3
    ................ucuguaaagcucaggagggauagc...       1   0   JCK-1
    ................ucuguaaagcucaggagggauagc...       1   0   LT-1
    ..................uguaaagcucaggaggga...           6   0   JT-3
    ......................aagcucaggagggauagc...       1   0   LCK-3
    ......................aagcucaggagggauagc...       1   0   LT-3
    ......................aagcucaggagggauagc...       2   0   LT-2
    ......................aagcucaggagggauagc...       3   0   LT-1
    ......................aagcucaggagggauagcgc...     1   0   LT-3
    ......................aagcucaggagggauagcgc...     2   0   JT-2
    ......................aagcucaggagggauagcgc...     7   0   JT-3
    ......................aagcucaggagggauagcgc...     1   0   LT-1
    ......................aagcucaggagggauagcgc...     1   0   LT-2
    ......................aagcucaggagggauagcgcc...    38  0   JCK-1
    ......................aagcucaggagggauagcgcc...    59  0   JCK-2
    ......................aagcucaggaggUauagcgcc...    3   1   JCK-2
    ......................aagcucaggagggauagcgcc...    2   0   LCK-2
    ......................aagcucaggagggauagcgcc...    30  0   LT-1
    ......................aagcucaggagggauagcgcc...    7   0   LCK-3
    ......................aagcucaggagggauagcgcc...    37  0   LT-3
    ......................aagcucaggagggauagcgcU...    1   1   LT-3
    ......................aagcucaggagggauagcgcc...    12  0   LT-2
    ......................aagcucaggagggauagcgcc...    56  0   JCK-3
    ......................aagcucaggagggauagcgccA...   1   1   LCK-3
    .......................agcucaggagggauagcgcc...    1   0   JCK-3
    .......................agcucaggagggauagcgcc...    2   0   LT-3
    .......................agcucaggagggauagcgcc...    2   0   JCK-2
    .......................agcucaggagggauagcgcc...    2   0   JCK-1
    .......................agcucaggagggauagcgccg...   1   0   JCK-2
    .......................agcucaggagggauagcgccA...   1   1   LT-3
```

```
                      Mature                                                                        Star
aaacaguaagugagaaucuguaaagcucaggagggauagcgccgugcgaacaaguuugguuuuaauuucauguuguuuugcuuuuuuggucgccauuggcgcuaucuauccugaguuuuauagguucuucuuacuacauguauaucucugguacucaugcaguacaguuuucaguucacucagcuggagguauuguauuuggcuccuugguaugcaacaugauacuacauauaucuguuaaguaauuuac
.......................agcucaggagggauagcgccg..............................................................................................................................................................................   1   0   LT-3
..................................................................................................gcgcuaucuauccugaguuuu...................................................................................................................................   5   0   JCK-3
..................................................................................................gcgcuaucuauccugaguuuu...................................................................................................................................   4   0   JCK-2
..................................................................................................gcgcuaucuauccugaguuuu...................................................................................................................................   3   0   LT-3
..................................................................................................gcgcuaucuauccugaguuuu...................................................................................................................................   2   0   JCK-1
...................................................................................................cgcuaucuauccugaguu.....................................................................................................................................   3   0   LCK-3
...................................................................................................cgcuaucuaucAugaguu.....................................................................................................................................   1   1   JCK-3
...................................................................................................cgcuaucuauccugaguu.....................................................................................................................................   16  0   LT-3
...................................................................................................cgcuaucuauccugaguu.....................................................................................................................................   10  0   LT-1
...................................................................................................cgcuaucuauccugaguu.....................................................................................................................................   4   0   JCK-2
...................................................................................................cgcuaucuauccugaguu.....................................................................................................................................   2   0   JCK-3
...................................................................................................cgcuaucuauccugaguu.....................................................................................................................................   15  0   LT-2
...................................................................................................cgcuaucuauccugaguu.....................................................................................................................................   3   0   JCK-1
...................................................................................................cgcuaucuauccugaguuu....................................................................................................................................   3   0   JCK-3
...................................................................................................cgcuaucuauccugaguuu....................................................................................................................................   2   0   LT-2
...................................................................................................cgcuaucuauccugaguuu....................................................................................................................................   6   0   LT-3
...................................................................................................cgcuaucuauccugaguuu....................................................................................................................................   4   0   LCK-3
...................................................................................................cgcuaucuauccugaguuu....................................................................................................................................   2   0   JCK-2
...................................................................................................cgcuaucuauccugaguuu....................................................................................................................................   5   0   JCK-1
...................................................................................................cgcuaucuauccugaguuu....................................................................................................................................   2   0   LT-1
...................................................................................................cgcuaucCauccugaguuuu...................................................................................................................................   14  1   LT-3
...................................................................................................cgcuaucuauccugaguuuC...................................................................................................................................   1   1   LT-3
...................................................................................................cgcuaucCauccugaguuuu...................................................................................................................................   9   1   JCK-3
...................................................................................................cgcuaucuauccugaguuuA...................................................................................................................................   2   1   LT-1
...................................................................................................cgcuaucuauccugaguuuu...................................................................................................................................   14  0   LT-3
...................................................................................................cgcuaucuauccugCguuuu...................................................................................................................................   1   1   JCK-3
...................................................................................................cgcuaucuauccugaguuuu...................................................................................................................................   12  0   JCK-1
...................................................................................................cgcuaucuauccugaguuuu...................................................................................................................................   5   0   LT-1
...................................................................................................cgcuaucuauccugaguuuA...................................................................................................................................   1   1   LCK-3
...................................................................................................cgcuaucuauccugaguuuu...................................................................................................................................   9   0   LT-2
...................................................................................................cgcuaucCauccugaguuuu...................................................................................................................................   18  1   JCK-2
...................................................................................................cgcuaucuauccugaguuuu...................................................................................................................................   20  0   JCK-3
...................................................................................................cgcuaucCauccugaguuuu...................................................................................................................................   7   1   LT-2
...................................................................................................cgcuaucuauccugaguuuu...................................................................................................................................   3   0   JT-3
...................................................................................................cgcuaucCauccugaguuuu...................................................................................................................................   8   1   LT-1
...................................................................................................cgcuaucuauccugaguuuu...................................................................................................................................   23  0   JCK-2
...................................................................................................cgcuaucuauccugaguuuA...................................................................................................................................   2   1   LT-2
...................................................................................................cgcuaucCauccugaguuuu...................................................................................................................................   6   1   LCK-3
...................................................................................................cgcuaucCauccugaguuuu...................................................................................................................................   5   1   JCK-1
...................................................................................................cgcuaucuauccugaguuuu...................................................................................................................................   3   0   JT-2
...................................................................................................cgcuaucuauccugaguuuua..................................................................................................................................   3   0   JCK-1
...................................................................................................cgcuaucuauccugaguuuua..................................................................................................................................   1   0   LT-1
...................................................................................................cgcuaucuauccugaguuuua..................................................................................................................................   7   0   LCK-3
...................................................................................................cgcuaucuauccugaguuuuU..................................................................................................................................   1   1   JCK-1
...................................................................................................cgcuaucuauccugaguuuua..................................................................................................................................   3   0   JCK-2
...................................................................................................cgcuaucuauccugaguuuuU..................................................................................................................................   1   1   JCK-3
...................................................................................................cgcuaucuauccugaguuuuU..................................................................................................................................   1   1   LT-3
...................................................................................................cgcuaucuauccugaguuuuU..................................................................................................................................   2   1   JCK-2
....................................................................................................gcuaucCauccugaguuuu...................................................................................................................................   1   1   LT-1
....................................................................................................gcuaucuauccugaguuuu...................................................................................................................................   2   0   JCK-1
....................................................................................................................................acuacauguauaucucugguacu...............................................................................................   1   0   LT-1
```
